# Supplementary material for: Antibody to Endogenous Cardiotonic Steroid Reverses Vascular Fibrosis and Restores Vasorelaxation in Chronic Kidney Disease
Source: Int J Mol Sci. 2024 Aug 15;25(16):8896. doi: 10.3390/ijms25168896 (PMC11354990; doi:10.3390/ijms25168896)
Supplement: Supplementary file 1 [file ijms-25-08896-s001.zip › ijms-3077672-supplementary.pdf]

International Journal of
Molecular Sciences

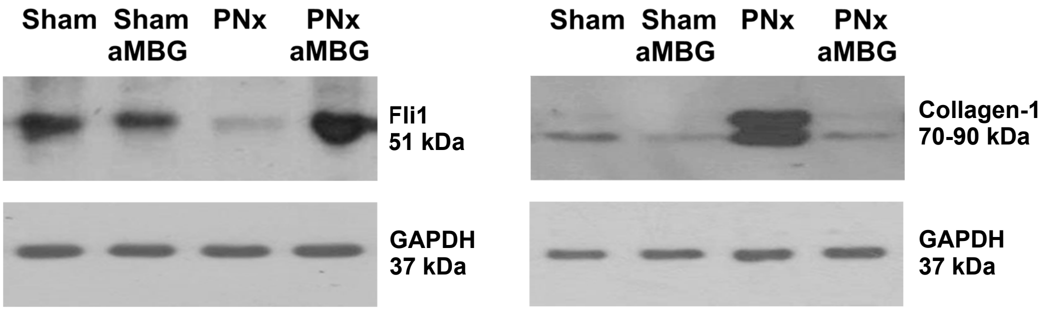

Supplementary Figure S1. Effect of aMBG antibodies on protein levels of Fli1 and Collagen-1 in thoracic aortae of Sham and PNx rats administered with anti-MBG antibodies (representative Western blots images of 4 assays).
